# P-1212. Effects of Quorum Sensing–Interfering Agents, Including Macrolides and Furanone C-30, and an Efflux Pump Inhibitor on Nitrosative Stress Sensitivity in Pseudomonas aeruginosa

**DOI:** 10.1093/ofid/ofaf695.1405

**Published:** 2026-01-11

**Authors:** Shin Suzuki, Yuji Morita, Shota Ishige, Kiyohiro Kai, Kenji Kawasaki, Kazuyuki Matsushita, Kohei Ogura, Tohru Miyoshi-Akiyama, Takeshi Shimizu

**Affiliations:** Chiba University /Chiba University Hospital, Chiba, Chiba, Japan; Meiji Pharmaceutical University, Kiyose, Tokyo, Japan; Chiba University, Chiba, Chiba, Japan; Chiba University, Chiba, Chiba, Japan; Chiba University Hospital, Chiba, Chiba, Japan; Chiba University Hospital, Chiba, Chiba, Japan; Kyoto University, Uji, Kyoto, Japan; National Center for Global Health and Medicine, Shinjuku, Tokyo, Japan; Chiba University, Chiba, Chiba, Japan

## Abstract

**Background:**

Long-term administration of certain macrolides is efficacious in patients with persistent pulmonary *Pseudomonas aeruginosa* infection, despite the limited clinically achievable concentrations far below their MICs. An increase in the sub-MIC of macrolide exposure-dependent sensitivity to nitrosative stress is a typical characteristic of *P. aeruginosa*. However, a few *P. aeruginosa* clinical isolates do not respond to sub-MIC of macrolide treatment. Therefore, we examined the effects of sub-MIC of macrolide on the sensitivity to nitrosative stress together with an efflux pump inhibitor (EPI). In addition, due to macrolides interfering with quorum sensing (QS), we confirmed the effect of QS-interfering agents furanone C-30 (C-30).

**Figure:**
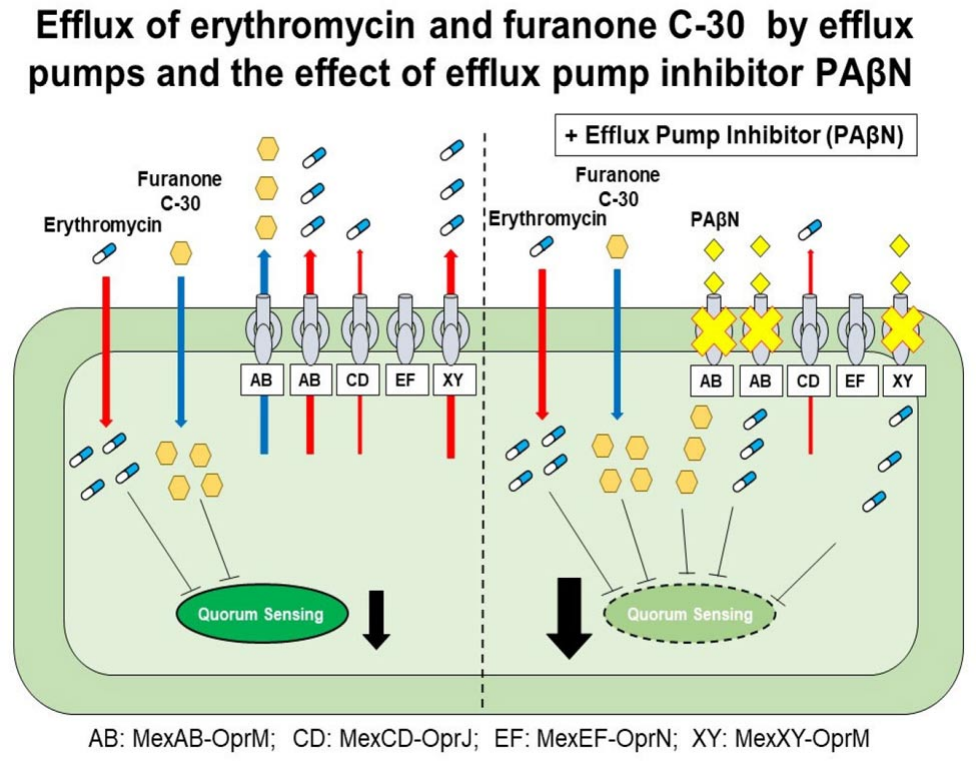
Efflux of erythromycin and furanone C-30 by efflux pumps and the effect of efflux pump inhibitor PAβN

**Methods:**

Nitric oxide (NO) sensitivity assays were performed using *P. aeruginosa* PAO1, efflux pump-mutant strains, and 11 clinical isolates. Strains were reacted with erythromycin (EM) 10 µg/mL as macrolide, C-30 10 µM as QS inhibitor, phenylalanine arginyl β-naphthylamide (PAβN) 10 µg/mL as EPI, and DETA NONOate 100 µM as NO donor, respectively. The number of viable bacteria was determined using bacterial plate count.

**Results:**

The sensitivity to nitrosative stress increased when using EPI in treatment with sub-MIC of macrolide, suggesting that the efflux pump was involved in inhibiting the sub-MIC of macrolide effect (at least n=3, p< 0.01). Analysis using efflux pump-mutant *P. aeruginosa* revealed that MexAB-OprM, MexXY-OprM, and MexCD-OprJ are factors in reducing the sub-MIC of macrolide effect (at least n=3, p< 0.01). We also demonstrated that the QS-interfering agent C-30 producing greater sensitivity to NO stress than EM, and the effect of C-30 was decreased by overproduction of MexAB-OprM. To investigate whether the increase in the QS-interfering agent exposure-dependent sensitivity to nitrosative stress is characteristic of *P. aeruginosa* clinical isolates, we examined the viability of *P. aeruginosa* treated with NO. As a result, in 9 of 11 strains, C-30 was highly effective at reducing cell viability, and treatment with both C-30 and PAβN was sufficiently effective against the remaining isolates.

**Conclusion:**

The combination of a QS-interfering agent and an EPI could be effective in treating *P. aeruginosa* infections.

**Disclosures:**

All Authors: No reported disclosures

